# Prognostic relevance of early clinical and laboratory findings in immune-mediated thrombotic thrombocytopenic purpura

**DOI:** 10.1016/j.rpth.2025.102974

**Published:** 2025-07-18

**Authors:** Atsushi Hamamura, Kazuya Sakai, Toshiki Mushino, Yasunori Ueda, Yoshiyuki Ogawa, Hiroyuki Noguchi, Akinao Okamoto, Hideo Yagi, Takehiko Mori, Masanori Matsumoto

**Affiliations:** 1 Department of Blood Transfusion Medicine, Nara Medical University, Kashihara, Japan; 2 Department of Hematology, Institute of Science Tokyo, Bunkyo-ku, Japan; 3 Department of Hematology/Oncology, Wakayama Medical University, Wakayama, Japan; 4 Community Medical Support Center, Wakayama Medical University, Wakayama, Japan; 5 Department of Hematology/Oncology, Hemapheresis Center, Kurashiki Central Hospital, Kurashiki, Japan; 6 Department of Hematology, Gunma University Graduate School of Medicine School of Medicine Faculty of Medicine, Maebashi, Japan; 7 Department of Hematology, Maebashi Red Cross Hospital, Maebashi, Japan; 8 Department of Hematology, Fujita Health University, Toyoake, Japan; 9 Department of Hematology and Oncology, Nara Prefecture General Medical Center, Nara, Japan; 10 Department of Hematology, Nara Medical University, Kashihara, Japan

**Keywords:** ADAMTS-13, cardiac symptoms, neurologic symptoms, thrombotic thrombocytopenic purpura, von Willebrand factor

## Abstract

**Background:**

Immune-mediated thrombotic thrombocytopenic purpura (iTTP) is a life-threatening condition caused by a severe deficiency of a disintegrin and metalloproteinase with thrombospondin type 1 motif 13 due to autoantibodies. Despite modern treatments, including therapeutic plasma exchange, immunosuppression, and rituximab, early mortality—often due to cardiac and neurologic events—remains a concern.

**Methods:**

We analyzed data from 125 patients between 2010 and 2023 in the Japanese thrombotic thrombocytopenic purpura (TTP) registry, examining demographics, electrocardiogram and transthoracic echocardiography findings, and neurologic symptoms. Troponin I was measured. Outcomes were categorized as survivors, TTP-related deaths, and non–TTP-related deaths.

**Results:**

Of the 125 patients, 15 died, with 5 deaths directly related to iTTP. Early cardiac findings and neurologic symptoms were not significant predictors of mortality. However, elevated lactate dehydrogenase levels and reduced von Willebrand factor multimer indices correlated with poorer prognosis. Patients with myocardial hypokinesis finally recovered their condition during the course of treatment. No patient treated with caplacizumab died during the observation period.

**Conclusions:**

These findings suggest that early cardiac and neurologic symptoms may not be definitive predictors of iTTP-related death. Instead, extremely high lactate dehydrogenase levels indicated a worse prognosis, highlighting the need for targeted monitoring and interventions in high-risk cases.

## Essentials

• In iTTP, clinical findings related to heart function at onset may be related to disease mortality.
• We retrospectively analyzed the prognostic impact of these clinical findings.
• Clinical findings did not predict disease mortality in our cohort.
• Despite signs of low blood supply, timely therapy remains effective for restoring heart function.

## 1 Introduction

Thrombotic thrombocytopenic purpura (TTP) is a life-threatening thrombotic disease characterized by a severe deficiency in the activity of a specific von Willebrand factor (VWF)-cleaving protease, a disintegrin-like metalloproteinase with thrombospondin type 1 motif 13 (ADAMTS-13) [1]. This deficiency leads to the accumulation of ultralarge VWF multimers, which form platelet-rich microthrombi, causing mechanical hemolysis and thrombocytopenia. TTP can be congenital, resulting from *ADAMTS-13* gene mutations, or more commonly acquired as immune-mediated TTP (iTTP), where autoantibodies inhibit ADAMTS-13 activity. Clinical features typically include hemolytic anemia, severe thrombocytopenia, and multiorgan dysfunction, often affecting the cardiac and neurologic systems owing to microvascular thrombosis [2].

Since the efficacy of therapeutic plasma exchange (TPE) was established in 1991, the mortality rate with iTTP has significantly declined from 90% to approximately 10% to 20% [3,4]. Additional immunosuppressive therapies, including corticosteroids and rituximab, have further improved patient outcomes [5]. However, a subset of patients experience early mortality, often within the first 2 weeks of therapy, thus suggesting that early mortality remains a critical issue [6].

Cardiac complications in patients with iTTP are well-documented, with reports of myocardial ischemia, arrhythmias, and even sudden cardiac death [6,7]. Histopathologic findings in patients who experienced early mortality during treatment often show microthrombi and ischemic changes in the myocardium [6,8]. These observations highlight the need to determine whether early cardiac symptoms, electrocardiographic (ECG) abnormalities, transthoracic echocardiography (TTE) findings, and cardiac troponins predict fatal outcomes [9]. Previous studies have suggested a correlation between cardiac involvement and mortality [7]; however, these findings are controversial.

The introduction of TPE, immunosuppressive therapy, and adjunctive therapies may influence the impact of early cardiac findings on the prognosis (or outcome). Similarly, neurologic symptoms are common in iTTP; they range from mild symptoms, such as headache, to severe impairments, including confusion, focal neurologic deficits, seizures, and coma [2].

Mild symptoms such as headache or dizziness are sometimes overlooked in clinical situations. Alwan et al. [10] showed that a lower Glasgow Coma Scale (GCS) score of <15 was associated with poor prognosis. Regarding VWF multimeric analysis, Béranger et al. [11] demonstrated a relationship between neurologic manifestations and the loss of ultralarge VWF multimers. They suggested a pathophysiological link between VWF multimeric patterns and neurologic involvement [11]. However, the prognostic significance of these neurologic symptoms/findings and their mechanistic connection to VWF multimeric profiles in the current management era remain unclear.

We examined a multicentre registry of patients diagnosed with iTTP between 2010 and 2023 to determine whether early cardiac symptoms, ECG and TTE findings, and cardiac enzymes (troponin T/I) can predict prognosis in the post-TPE era. Additionally, we explored the relationship between neurologic findings and large VWF multimer levels. By integrating clinical, laboratory, and imaging data, we enhanced our understanding of early prognostic factors for iTTP and informed targeted therapeutic interventions.

## Materials and Methods

### 2.1 Patient enrolment

Nara Medical University is the largest reference center for TTP in Japan; it measures ADAMTS-13 levels in citrate plasma samples from suspected patients nationwide. Patients diagnosed with iTTP between 2010 and 2023 were enrolled. The diagnosis of iTTP required severe ADAMTS-13 activity deficiency (<10%), detectable ADAMTS-13 inhibitors, and laboratory evidence of hemolytic anemia and thrombocytopenia.

Data were collected through standardized digital questionnaires distributed to each institution. We gathered demographic information, including age, sex, presenting symptoms, laboratory parameters, treatment details, and outcomes. We queried clinicians about the presence of cardiac symptoms at diagnosis, including cardiac pain, dyspnea, or other cardiopulmonary complaints. Neurologic manifestations were categorized as headaches, altered mental status (eg, confusion or personality changes), sensory disturbances, focal signs, consciousness disturbances, or seizures. The patients were classified into 3 groups: survivors, TTP-related deaths, and non–TTP-related deaths. All participating institutions obtained approval from their respective research ethics committees for data sharing, and the study was conducted in accordance with the principles of the Declaration of Helsinki. Patients were considered to have provided informed consent unless they opted out of the study.

### 2.2 ECG and echocardiography analysis

For cardiac evaluation, ECG and TTE findings at admission or early during the treatment course were collected. ECG findings were classified using the modified Minnesota Code Classification System, with an additional category for QT prolongation. TTE findings were assessed according to the American Society of Echocardiography guidelines and included the evaluation of left ventricular ejection fraction (EF), valvular regurgitation, pericardial effusion, and regional wall motion abnormalities [12].

### 2.3 Biochemical measurements

Blood samples were collected at diagnosis or early during the treatment course. Citrated plasma was prepared and stored at −30 °C until further analysis. Troponin I level was measured using commercially available ELISA kits (Human Cardiac Troponin I SimpleStep ELISA Kit; Abcam). The cutoff level for troponin I in the ELISA kit was defined as 26.2 pg/mL, according to the cutoff level determined in Japan. All standard curves of the ELISA kits were depicted with the 4-parameter logistic curve, whereas the estimated levels of these data were calculated using the inverse functions of 4-parameter logistic curve. The VWF antigen level was measured using the CN-3000 system (Sysmex).

### 2.4 ADAMTS-13 activity and inhibitor assay

ADAMTS-13 activity and inhibitor levels were measured using an in-house ADAMTS-13 act-ELISA. This assay detects cleavage products of the minimal substrate peptide (VWF73), which is fused with glutathione S-transferase and a 6xHis tag and is captured by a specific monoclonal antibody (N10) [13].

### 2.5 VWF multimeric analysis

VWF multimeric analysis was performed on citrated plasma samples [14]. Samples were electrophoresed at 4 °C for 19 hours on a 1.4% agarose gel (SeaKem Gold Agarose; Ronza) with a 0.8% stacking gel (SeaKem HGT Agarose; Ronza). The separated VWF multimers were transferred to polyvinylidene difluoride membranes and immunoblotted using rabbit anti–human VWF polyclonal immunoglobulin G conjugated to horseradish peroxidase (Polyclonal Rabbit Anti–Human Von Willebrand Factor; Agilent). Densitometric analysis was performed using Fiji (ImageJ) [15], and large VWF multimers were defined as those corresponding to the 11th peak or higher from the lowest molecular weight fraction (Supplementary Figure S1). The large VWF multimer index was calculated as the ratio of the area under the curve of large multimers in the patient sample relative to that of pooled normal plasma [16].

### 2.6 Statistical analysis

TTP-related deaths were defined as continuous variables, summarized as medians with IQRs, and compared among groups using the Kruskal–Wallis test. Categorical variables were compared using the chi-square test or Fisher exact test, as appropriate. Multivariate regression analysis was performed to assess the relationship between the large VWF multimer index and neurologic findings; each finding was treated as categorical data. Statistical significance was set at *P* < .05, and statistical analyses were conducted using Python version 3.11.10 (The Python Software Foundation).

## 3 Results

### 3.1 Patient characteristics

**Figure 1:**
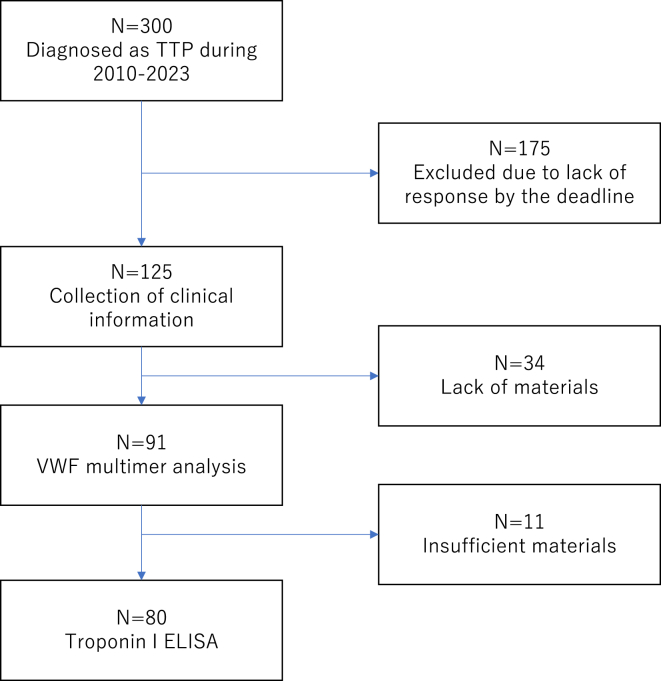
Study flowchart. A total of 300 cases were diagnosed with immune-mediated thrombotic thrombocytopenic purpura (iTTP) during the study period. Of these, 175 cases were excluded from further analysis due to lack of response by the deadline. Clinical information was gathered from the remaining 125 cases, and we performed a von Willebrand factor (VWF) multimer analysis and ELISA assay. TTP, thrombotic thrombocytopenic purpura.

Three hundred patients were diagnosed with iTTP at each collaborative institute during the study period, and 125 patients with confirmed iTTP were eligible for this study (Figure 1). Female patients were predominant (*n* = 68), with a median age of 54 years (range, 8-88 years). Among these 125 patients, 16 experienced a relapse during the study period, and 15 died (Table 1). Five of the deaths were directly attributable to iTTP-related events: 4 from sudden cardiac death and 1 from alveolar hemorrhage, which is an uncommon feature in iTTP (Table 2). The patient who died from alveolar hemorrhage also had cerebral infarction at acute incident. The remaining 10 patients died from causes unrelated to iTTP, including aspiration pneumonia (*n* = 3), malignant neoplasm (*n* = 2), and severe sepsis (*n* = 2). The characteristics of non–TTP-related deaths are detailed in Supplementary Table S1. Caplacizumab was administered for 16 patients.

**Table 1:** Demographics.

Characteristic	Value, *N* = 125 (%)
Sex	
Male	57
Female	68
Age (y), median (minimum-maximum)	54.0 (8-88)
Chest findings	13 (10)
Neurologic findings indicated	106/112 (95)
Electrocardiogram findings indicated	49/100 (49)
Transthoracic echocardiogram findings indicated	18/32 (56)
Troponin positive	40/56 (71)
B-type natriuretic peptide positive	51/62 (82)
Caplacizumab used	16 (13)
Relapse or exacerbation	16 (13)
TTP-related death	5 (4)
Non–TTP-related death	10 (8)

TTP, thrombotic thrombocytopenic purpura.

**Table 2:** Details of the patients died from immune-mediated thrombotic thrombocytopenic purpura.

ID	Age (y)	Sex	Hb (g/dL)	Platelet (109/L)	LDH (U/mL)	T-Bilirubin (mg/dL)	Creatinine (mg/dL)	Troponin I (ng/L)	Chest symptoms	ECG findings	TTE findings	ADAMTS-13 activity (%)	Inhibitor (BU/mL)	No. of PE	Survival (d)	Cause of death
1	66	M	14.6	7	2737	6.5	3.36	6.2	None	AF	NA	<0.5	2.9	4	4	Sudden cardiac death
2	69	F	11.2	16	1637	6.5	0.79	2376	None	PAC	NA	0.8	4.8	1	6	Sudden cardiac death
3	49	F	8.9	19	2966	2.1	2.17	36	None	Sinus tachycardia	NA	<0.5	5.1	9	12	Sudden cardiac death
4	62	M	9.5	4	2454	6.3	0.88	NA	None	Sinus rhythm	NA	<0.5	3.6	7	12	Alveolar hemorrhage
5	75	F	9.7	10	2560	4.1	1.12	194.6	None	Sinus rhythm	None	<0.5	1.6	0	1	Sudden cardiac death

ADAMTS-13, a disintegrin-like metalloproteinase with thrombospondin type 1 motif 13; AF, atrial fibrillation; ECG, echocardiogram; LDH, lactate dehydrogenase; NA, not available; PAC, premature atrial contraction; PE, pulmonary embolism; TTE, transthoracic echocardiography.

### 3.2 Cardiac and neurologic findings

Cardiac symptoms at diagnosis were documented in 13 patients, including exertional dyspnea (*n* = 6), cardiac pain (*n* = 4), cardiac discomfort (*n* = 2), and unknown details (*n* = 1). All patients who reported cardiac symptoms at disease onset survived. Neurologic data were documented for 112 patients (Table 1). Positive findings were observed in 106 patients (94.6%), including focal signs (*n* = 34), altered mental status (*n* = 11), consciousness disturbances (*n* = 51), seizures (*n* = 6), headaches (*n* = 18), and sensory disturbances. Other findings from these categories were observed in 9 patients, and the major finding was vertigo or dizziness (data not shown).

### 3.3 ECG and TTE findings

**Figure 2:**
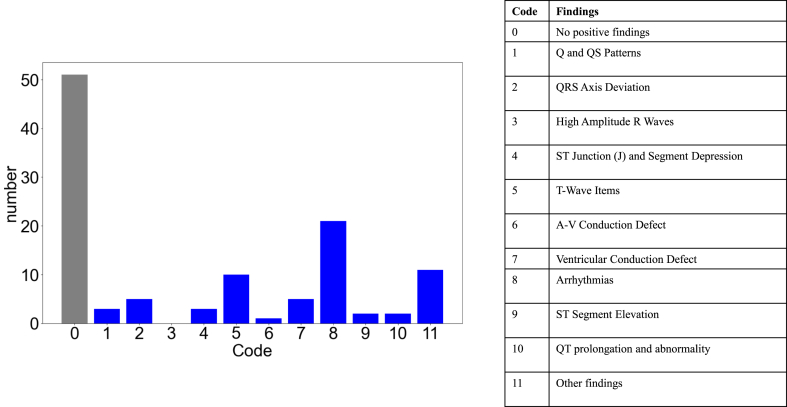
Classification of echocardiogram (ECG) findings. Of 100 patients, 49 findings were documented. The numbers on the x-axis correspond to the numbers in the modified Minnesota code classification system (as illustrated in the table to the right, with an additional category for QT prolongation). The most commonly detected finding was arrhythmia, comprising sinus tachycardia and bradycardia.

ECG data were available for 100 patients, and abnormalities were detected in 49 patients (Table 1). The most frequently detected ECG finding was arrhythmia (*n* = 21), followed by T-wave abnormalities (*n* = 10). Other findings included axial deviations and low amplitudes (*n* = 11) (Figure 2). No ST changes and wide QRS, and fatal arrythmia were not observed, which suggest cardiac ischemia and changes.

TTE was performed in 32 patients, and significant findings (such as EF reduction, valvular regurgitation, and pericardial effusion) were observed in 18 patients (Table 1). Moderate or more valvular regurgitation was observed in 1 patient. The median EF was 64.05% (IQR, 57.9%-71.0%). Pericardial effusion was observed in 8 patients (Supplementary Table S2). Hypokinesis was indicated in 4 patients, whereas 1 patient had severe diffuse hypokinesis but recovered wall motion during therapy. One patient who succumbed to iTTP underwent TTE; however, no findings were observed.

However, none of the ECG or TTE findings showed a statistically significant correlation with TTP-related mortality (*P* = .85 and .865, respectively). Three of the 5 TTP-related deaths had ECG abnormalities; however, these abnormalities were nonspecific and did not predict imminent fatal arrhythmia.

### 3.4 Troponin measurement

Troponin T or I levels were assessed in 56 patients at each institute (Table 1). Troponin positivity was detected in 40 patients at each institute. However, elevated troponin levels did not distinguish survivors from those who died of TTP-related events. Similarly, troponin I measured in 80 patients at our institute using stored plasma samples, showed no significant differences in the median levels among the 3 outcome groups (*P* = .17). The median troponin I level measured in our institute was 36.0 pg/mL (IQR, 8.25-103 pg/mL). No significant difference was observed in the positivity rate of troponin I among the 3 groups based on ELISA values measured at our institute (*P* = .16). Further, when the cutoff level for troponin I was set at 250 pg/mL as reported previously [17], the results remained nonsignificant (*P* = .55). Ten patients were positive for troponin I at aforementioned threshold, 8 of whom were in the survivors group. The remaining 2 patients died from TTP or aspiration pneumonia.

### 3.5 ADAMTS-13 parameters and therapies

All patients in the TTP-related death group had ADAMTS-13 activity of <10%, and inhibitors were >1.0 BU/mL. None of the patients in this group received caplacizumab (as these cases occurred before its introduction in Japan), whereas 15 survivors received caplacizumab. This observation suggests that anti-VWF therapies may improve early survival; however, more data and longer follow-up periods are required.

### 3.6 VWF multimeric analysis and VWF antigen measurement

**Figure 3:**
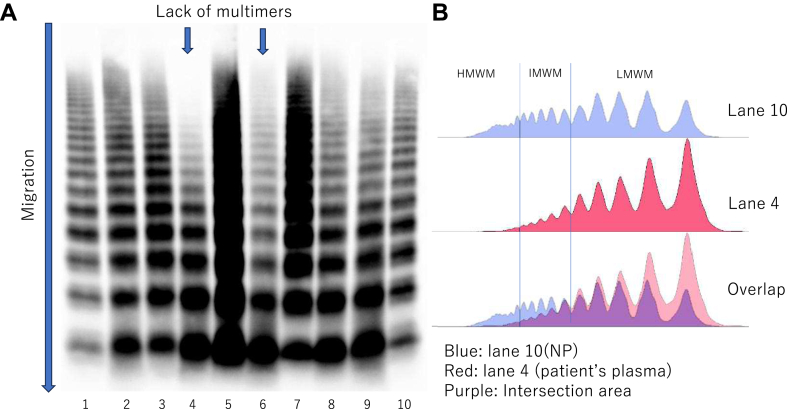
Lack of von Willebrand factor (VWF) multimeric bands. (A) In VWF multimeric analysis, lack of high-molecular-weight VWF multimers (HMWM) was observed in some patients with immune-mediated thrombotic thrombocytopenic purpura. Lanes 4 and 6 lack HMWM band compared with normal plasma (lanes 1 and 10). (B) Analyzing with densitometry, the curve based on patient’s plasma have more low-molecular-weight VWF multimers (LMWM) and less HMWM than the curve based on normal plasma. IMWM, intermediate-molecular-weight VWF multimers.

A VWF multimer analysis was performed using data from 91 patients. Five cases lacked high-molecular-weight multimers (Figure 3, only 1 case is shown). The mean large VWF multimer index was 56.7%. Patients who died of TTP-related events tended to have a large VWF multimer index of 39.6%, which was lower than the 51.3% of the survivors, whereas patients who died of non–TTP-related causes tended to have a large multimer index of 82.0% (Table 3). Evaluation of the correlation coefficient between the presence of cardiac symptoms and the multimer index showed no significant correlation (*r* = −0.06). Neurologic findings did not correlate with a large VWF multimer index in the multivariate regression analysis. All covariates in the multiple regression analysis did not reach significance levels (Supplementary Table S3). VWF antigen level was measured and the median level was 184.9% (IQR, 134.4%-266.6%). We found a positive correlation between the VWF antigen level and area under the curve (AUC) of the multimer ladder (*R* = 0.76; *P* < .001). The correlation between the VWF antigen level and multimer index was weaker than that between the VWF antigen level and AUC of multimers (*R* = 0.31; *P* < .005). For these patients, the blood types were as follows: A, *n* = 37; B, *n* = 21; O, *n* = 22; and AB, *n* = 10 cases.

**Table 3:** Characteristics of 3 groups.

Characteristic	Survivor (*n* = 110)	TTP-related death (*n* = 5)	Non–TTP-related death (*n* = 10)	*P*
Age (y)	51 (39-66.75)	66 (62-69)	77 (74.25-81.75)	<.001
Sex				.268
Male	48	2	7	
Female	62	3	3	
Positive chest findings (%)	12 (11)	0 (0)	1 (10)	.988
ECG findings, positive/entire (%)	42/86 (49)	3/5 (60)	4/9 (44)	.853
TTE findings, positive/entire (%)	16/41 (39)	0/1 (0)	2/7 (29)	.865
WBC (109/μL)	6.25 (4.68-8.70)	9.12 (8.80-10.25)	7.47 (5.20-8.05)	.058
Hemoglobin (g/dL)	7.95 (7.0-9.6)	9.7 (9.5-11.2)	8.4 (7.1-8.6)	.052
Platelets (109/μL)	11 (8-16)	10 (7-16)	10 (8-12)	.933
LDH (U/mL)	968 (657-1358)	2560 (2454-2737)	901.0 (574-1240)	<.005
T-bilirubin (mg/dL)	0.9 (1.925-4.7)	6.3 (4.1-6.5)	3.1 (1.9-3.3)	.119
Creatinine (mg/dL)	0.885 (0.63-1.15)	1.12 (0.88-2.17)	1.29 (1.06-1.32)	.024
ADAMTS-13 activity (%)	<0.5	<0.5	<0.5	.149
ADAMTS-13 inhibitor (BU/mL)	2.35 (1.0-4.45)	3.6 (2.9-4.8)	1.5 (0.85-3.175)	.333
TPE, No. of patients (%)	94 (85)	4 (80)	7 (70)	.504
TPE, No. of procedures (%)	9 (5.5-14)	5.5 (3.25-7.5)	14 (10-18)	.088
Troponin I (pg/mL)	7.573 (<4.4-74.52)	115.3 (28.53-740. 0)	32.61 (2.516-71.75)	.174
Large VWF multimer index (%)	51.3 (37.3-73.7)	39.6 (36.3-42.2)	82.0 (74.2-98.8)	.018
Follow-up period (d)	1312 (397.5-2410)	6 (4-12)	77 (45-892.5)	a

Values in brackets are IQR (25%-75%). Red colored fonts mean the statistical significance.

ADAMTS-13, a disintegrin-like metalloproteinase with thrombospondin type 1 motif 13; BU, Bethesda unit; ECG, electrocardiogram; LDH, lactate dehydrogenase; TPE, therapeutic plasma exchange; TTE, transthoracic echocardiogram; VWF, von Willebrand factor; WBC, white blood cell.

a *P* value was not estimated due to the nature of the comparisons.

### 3.7 Survival analysis and statistical findings

The median follow-up was 1026 days. Among the 3 groups, significant differences were observed in age (median value in group of survivor, 51 years; TTP-related death, 66 years; non–TTP-related death, 77 years; *P* < .001), lactate dehydrogenase at presentation (median value in group of survivor, 968 U/mL; TTP-related death, 2560 U/mL; non–TTP-related death, 1166 U/mL; *P* < .005), creatinine level (median values in the survivors, TTP-related death, and non–TTP-related death groups were 0.885, 1.12, and 1.29, mg/dL, respectively; *P* = .02), and large VWF multimer index (median value in group of survivor, 51.3; TTP-related death, 39.6; non–TTP-related death, 82.0; *P* = 0.018). Median value of follow-up period in group of survivor was 1312 days. Median values of survival days in group of TTP-related death and non–TTP-relate death were 6 and 77 days, respectively. Conversely, cardiac and neurologic findings and troponin level did not differ significantly among the groups (Table 3).

## 4 Discussion

Previous studies suggested that an elevated cardiac troponin concentration is associated with a poorer clinical outcome in iTTP [8,10,17]. Benhamou et al. [17] showed that a cardiac troponin I of > 250 ng/L resulted in a poor prognosis. In contrast, our study failed to detect any significant difference in baseline troponin values between the group of patients who died from TTP-related causes and the groups of patients who either survived or died from other causes. One potential explanation for this discrepancy is that the distribution of troponin values in our cohort differed fundamentally from that in the series by Benhamou et al. [17]; whereas 41% of their patients presented with a troponin I level of > 250 ng/L, the corresponding proportion in our data set was only 12.5%. Because troponin I is a sensitive marker of myocardial ischemia, these findings imply that the degree of myocardial injury at first presentation in our cohort was comparatively mild.

ECG provided concordant information. Notably, none of the patients exhibiting ST-segment deviation or fatal arrhythmias—abnormalities that would ordinarily denote severe, clinically overt myocardial ischemia. These observations reinforce the notion that, at onset, myocardial damage had not yet evolved to a stage detectable by routine ECG. Although sudden cardiac death in TTP results from fatal arrythmia caused by diffuse microthrombi in cardiac tissue [18], the absence of life-threatening ECG features in both the TTP-related and non–TTP-related death groups argues against this mechanism in our population.

Similarly, TTE yielded few relevant abnormalities. Most examinations were judged to be unremarkable, again supporting the premise that structural cardiac injury was limited at the time of diagnosis. Among the small subset of patients with pathologic findings, hypokinesis was identified in 4, of whom 1 had diffuse ventricular wall motion impairment. Remarkably, even these seemingly severe disturbances resolved during the subsequent course of treatment, highlighting the potential reversibility of myocardial involvement in acute TTP. Because TTP-associated thrombosis predominantly affects the microcirculation, the same principle applies to the myocardium. In a previous study involving a patient who experienced sudden death during an acute TTP episode, we documented diffuse microthrombi in the cardiac tissue [6]. Taken together, these and previous data suggest that timely therapeutic intervention can dissolve microvascular thrombi, thereby restoring myocardial perfusion and function. Consequently, the cases analyzed in this study may largely represent patients captured early in their disease course, when tissue injury was still reversible.

Neurologic manifestations constitute one of the classical clinical hallmarks of TTP, and their prognostic significance has been debated extensively. Alwan et al. [10] analyzed neurologic status using the GCS and reported a 9-fold increase in mortality risk when the GCS was ≤14 [10]. Béranger et al. [11] further observed a relationship between the severity of neurologic damages and the absence of high-molecular-weight VWF multimers (HMWM) [11]. In our multimer analysis, however, we did not find an association between HMWM deficiency and the presence, or severity, of neurologic signs. This discrepancy raises the possibility that the previous method used to grade neurologic impairment failed to capture the full spectrum of subtle but clinically relevant alterations. Refinement of neurologic assessment tools therefore remains an important objective for future prognostic studies in TTP.

The multimer index requires particular attention, because it differed across the 3 outcome categories examined—survivors, TTP-related deaths, and non–TTP-related deaths. Patients whose deaths were directly attributable to TTP displayed a trend toward a lower multimer index than that of survivors, whereas the opposing tendency emerged in patients who died from unrelated causes. Notably, plasma VWF Ag concentrations did not differ significantly among the groups (*P* = .117), and VWF Ag concentrations correlated strongly with the area under the densitometric curve of the multimer pattern (*R* = 0.76; *P* < .001). However, the correlation between the VWF Ag and multimer index was weaker than that between the VWF Ag and AUC of multimers (*R* = 0.31; *P* < .005). These observations imply that, in TTP-related deaths, a depressed multimer index primarily reflects a selective loss of HMWM and thus parallels disease activity [3]. In contrast, the unexpectedly high multimer index in non–TTP-related deaths may stem from the frequent presence of infections in our cohort, a circumstance known to increase both VWF release and proteolytic resistance.

The potential impact of caplacizumab is equally noteworthy. Although the principal aim of our study was not to evaluate therapeutic efficacy, it is striking that none of the patients who received caplacizumab succumbed to TTP-related early death. This finding is compatible with the early mortality–reducing effect suggested in previous trials. Coppo et al. [19] reported that, under caplacizumab therapy, an elevated troponin concentration no longer constitutes an independent risk factor for poor prognosis. However, given that caplacizumab has been on the market for only a limited period, careful long-term surveillance is still required to characterize its full safety and efficacy profile.

Several limitations of this work must be acknowledged. First, the study used a retrospective observational design and is therefore subject to inevitable selection bias. Second, interlaboratory variability in assay methodologies may have influenced quantitative results, particularly for biomarkers such as troponin and VWF multimers. Third, missing clinical data, inherent to retrospective chart review, may have attenuated the statistical power. Finally, the classification of deaths as TTP-related versus non–TTP-related relied upon the attending physician’s judgment, because autopsies were rarely performed; thus, misclassification cannot be entirely excluded.

In conclusion, this analysis did not detect clear associations between initial cardiac symptoms, neurologic manifestations, routine laboratory findings, and subsequent outcome in an early-presenting, relatively mild cohort of patients with iTTP. Nonetheless, the observation that even cases with echocardiographic evidence of diffuse hypokinesis recovered myocardial function during therapy underscores the substantial potential for reversibility of organ damage when diagnosis is made promptly and treatment is initiated without delay. These results therefore reinforce the necessity of early recognition and intensive management to maximize the likelihood of a favorable prognosis in TTP care.
