# Supplementary material for: Prognostic relevance of early clinical and laboratory findings in immune-mediated thrombotic thrombocytopenic purpura
Source: Res Pract Thromb Haemost. 2025 Jul 18;9(5):102974. doi: 10.1016/j.rpth.2025.102974 (PMC12356007; doi:10.1016/j.rpth.2025.102974)
Supplement: Supplementary Material [file mmc1.pdf]

Supplementary Table S1. Details of patients died of non-TTP deaths

ID	Age	Sex	Hb (g/dL)	Plt (10^9/L)	LDH (U/mL)	T-Bil (mg/dL)	Cre (mg/dL)	Troponin I	Chest symptoms	ECG findings	TTE findings	ADAMTS 13 activity (%)	Inhibitor (BU/mL)	Number of PE	Survival (days)	Cause of death
**1**	85	M	12.3	10	901	3.7	1.78	NA	None	None	AR, MR, TR	0	1	17	43	Aspiration pneumonia
**2**	74	F	7.1	19	521	1.9	2.56	NA	None	A-V Block, negative-T wave	MR, TR	8.3	<0.5	5	89	sepsis, DIC
**3**	82	M	11.5	12	574	2.6	1.29	NA	None	None	None	0.9	3.3	3	2518	Aspiration pneumonia
**4**	75	M	NA	NA	NA	NA	NA	Lower than sensitivity	None	None	None	0	1	22	1464	SMA syndrome
**5**	88	M	7.1	8	1509	3.3	0.95	3.75 ng/mL	None	None	None	0	0.7	5	159	Old age
**6**	68	F	8.4	12	1240	3.7	0.74	342 pg/mL	None	None	None	0	9.3	7	44	Anaphylactic shock
**7**	78	F	8.6	163	360	0.3	1.32	NA	None	Af	None	7.6	0.8	NA	11	Aspiration pneumonia
**8**	72	M	8.6	8	1934	3.1	1.06	NA	Chest discomfort	Abnormal Q wave	None	0	2	13	1137	Lung cancer
**9**	76	M	6.1	9	580	3.3	1.29	NA	None	None	None	0	2.8	14	48	Malignant lymphoma
**10**	81	M	7.4	8	1166	1.9	1.3	NA	None	Af	None	0	3.7	19	65	Sepsis

Abbreviations AR: aortic valve regurgitation, MR: mitral valve regurgitation, TR: tricuspid valve regurgitation, Af: atrial fibrillation, NA: not available, DIC: disseminated intravascular coagulation, SMA syndrome: superior mesenteric artery syndrome

Supplementary Table S2. Details of TTE findings

	N=32	
Positive findings	18	
Ejection fraction (median, IQR)	64.05% (57.9-71)	
Valvular regurgitation	Aorta	11
	Mitral	20
	Tricuspid	22
	Pulmonary	10
Pericardial effusion	8	
hypokinesis	4	

Supplementary Table S3. Multivariate Analysis of Neurological Findings for the large VWF multimer index

Symptoms	Coefficient	P value
Focal signs	0.0003	0.996
Mental disorders	-0.1	0.301
Consciousness disturbances	-0.0627	0.338
Seizure	0.0288	0.823
Headache	0.0144	0.877
Sensory disturbances	-0.0578	0.638
Other	-0.1425	0.174

Supplementary Figure S1. Calculation procedure of large VWF multimer index

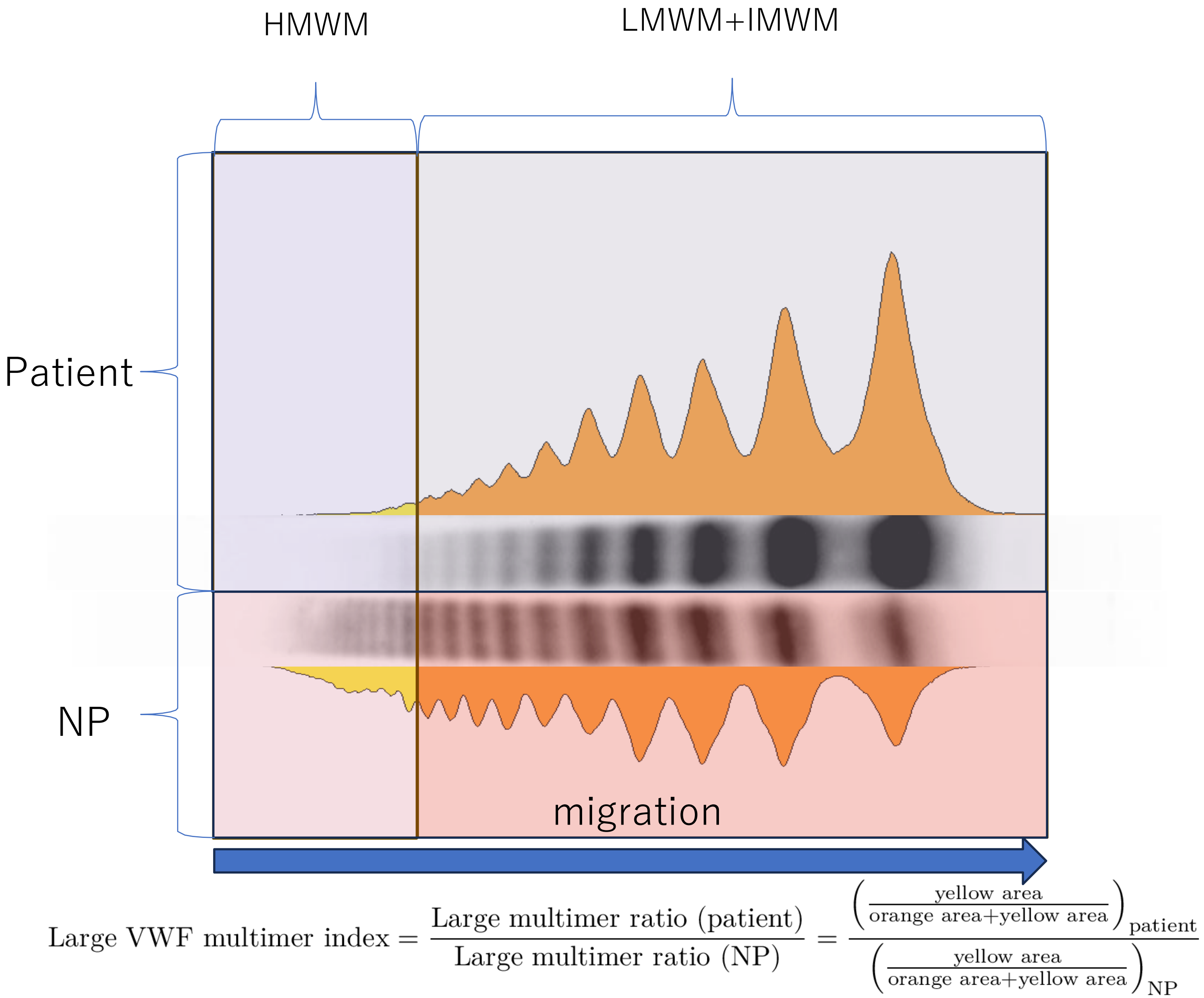

$$\text{Large VWF multimer index} = \frac{\text{Large multimer ratio (patient)}}{\text{Large multimer ratio (NP)}} = \frac{\left(\frac{\text{yellow area}}{\text{orange area+yellow area}}\right)_{\text{patient}}}{\left(\frac{\text{yellow area}}{\text{orange area+yellow area}}\right)_{\text{NP}}}$$

Abbreviations LMWM: low-molecular-weight-multimer; IMWM: intermediate-molecular-weight multimer; HMWM: high-molecular-weight multimer; NP: normal plasma
